# On ability of perch to colonize new waterbodies*—*indirect evidence and sticky facts. A Comment on: ‘Multiple lines and levels of evidence for avian zoochory promoting fish colonization of artificial lakes’ (2023), by Garcia *et al*.

**DOI:** 10.1098/rsbl.2023.0233

**Published:** 2023-09-13

**Authors:** Anti Vasemägi, Magnus Huss, Anna Gårdmark, Mikhail Ozerov

**Affiliations:** ^1^ Department of Aquatic Resources, Institute of Freshwater Research, Swedish University of Agricultural Sciences, Stångholmsvägen 2, 178 93 Drottningholm, Sweden; ^2^ Department of Aquatic Resources, Swedish University of Agricultural Sciences, Box 7018, 750 07 Uppsala, Sweden; ^3^ Biodiversity Unit, University of Turku, 20014 Turku, Finland; ^4^ Department of Biology, University of Turku, 20014 Turku, Finland

**Keywords:** bird-mediated colonization, freshwater, *Perca fluviatilis*

*Biol. Lett*. **19**: 20230533 (Published online 22 March 2023). (https://doi.org/10.1098/rsbl.2022.0533)

A recently published study [1] investigates the possible bird-mediated colonization of lakes by the Eurasian perch (*Perca fluviatilis*; henceforth: perch) using a ‘multiple lines and levels of evidence’ approach. The authors propose that their work supports avian zoochory based on a set of multidisciplinary approaches, including interviews, questionnaires, field surveys, population genetic analyses and literature. We argue, however, that the indirect indications provided by Garcia *et al.* [1] does not provide well-grounded support for the hypothesis of bird-mediated colonization, and we demonstrate that the presented results are ambiguous.

Garcia *et al.* [1] suggest that the co-occurrence of perch spawning with high waterfowl abundance in the littoral zone in freshwater lakes during spring can be considered as the first piece of evidence supporting avian zoochory. While we do not refute this co-occurrence, we note that the spatial overlap of fish and bird data used is highly limited and for the phenology, non-existing temporally. Fish were sampled in each of the 37 lakes once 2012–2019, whereas perch spawning was not observed but only predicted based on temperature in some (17) lakes and literature. The seasonality of the bird community, in contrast, was measured, not in the lakes, but in the centre of the study area and more than a decade earlier, in 1996–1998. It is also important to acknowledge that the majority of freshwater teleosts in the temperate zone reproduce during spring and early summer in shallow water. Thus, this claim is not specific to perch, and certainly, co-occurrence does not imply causation. Therefore, a future focus on dispersal of multiple fish species is warranted to better understand the factors and traits influencing colonization success.

The authors also incorrectly claim that Eurasian perch lays sticky or adhesive eggs (making them stick to waterfowl). In many fish species, including several percids, such as ruffe (*Gymnocephalus cernuus*) and pikeperch (*Sander lucioperca*), the outer layer of eggs facilitates egg attachment to aquatic substrates during spawning [2,3]. By contrast to the species mentioned above, perch eggs are not sticky when the egg strands come into a contact with water, nor are they sticky after fertilization [4]. Therefore, in order to provide sufficient water exchange and oxygen, the preferred spawning substrate for perch is submerged aquatic vegetation, roots and dead tree branches [5,6]. Because the egg ribbons are almost neutrally buoyant and may become entangled with submerged structures, such features may, at least partially, explain the misconception of sticky egg ribbons. The authors additionally suggest that since eggs of some fish species, such as herring (*Clupea harengus*), are common in waterfowl diets, the same may be true for perch. Several studies have, however, shown that the egg ribbons of both Eurasian perch and yellow perch (*Perca flavescens*) deter a variety of teleost (except Wels catfish) [7] and invertebrate predators because of unpalatability [8,9]. Furthermore, analysis of eggshells and the egg matrix in yellow perch has revealed a high concentration of a variety of potentially noxious components, including piperideine and nattectin [8]. Yet, despite substantial evidence of an unappealing taste of perch egg ribbons to predators, this and the unique feature of the gelatinous egg matrix was overlooked by Garcia *et al*. [1]. Therefore, we would like to highlight that while predation of perch eggs cannot be ruled out, the existing knowledge suggest that it is unlikely that perch eggs are actively consumed. On the other hand, the consumption of toxic plant species by waterfowl is rather common [10], which calls for experimental work testing whether perch egg ribbons are actively consumed by waterfowl and if perch embryos can survive the digestive system of different bird species [11].

The authors conclude that bird-driven colonization is a more likely process compared to human-mediated dispersal because of negligible illegal releases by anglers. However, their findings actually demonstrate that a stunning 20% of anglers have admittedly carried out illegal bucket releases of fish in the studied area, including releases of perch. Estimates based on voluntary surveys can also be expected to underestimate the actual figures, given that such releases are illegal and because access to 12 of the 37 lakes was prohibited. Thus, based on the presented evidence, fish stocking should not be ruled out as a realistic route for spread of fish to novel habitats. Furthermore, Garcia *et al*. [1] suggested that the observed weak positive correlation between geographical and genetic distances between populations (isolation by distance, IBD) supports bird zoochory. However, significant IBD patterns can be also generated by human-mediated dispersal and therefore IDB does neither corroborate nor refute the occurrence of bird zoochory. Finally, based on assignment analysis using 21 microsatellite markers, the authors detected eight individuals with multilocus genotypes that have a higher likelihood of originating from another population than where they were caught. However, misassignment does not necessarily mean that the individual originates from another population. It is well known that separating misassignments from real immigrants is challenging and depends on many factors, such as population divergence, the number of variable loci, marker variability, the size and completeness of the baseline dataset and the abundance of migrants [12]. Interestingly, a closer examination of the putative migrants revealed that, for some individuals, the likelihood of originating from a foreign or local population were rather similar. Thus, an alternative and perhaps more parsimonious explanation is that these individuals may represent misassignments rather than true migrants. Furthermore, additional simulations using the ONCOR software [13] (200 simulated samples per population, 1000 replications, electronic supplementary material, table S1) revealed that, while the mean assignment accuracy is high (mean of 99.49%), for some populations the accuracy is lower (i.e. 94.2, 96.5, 98.4%). Thus, misassignments in large datasets occur because of imperfect assignment and do not necessarily reflect migration events. All things considered, we would like to emphasize that the accurate detection of migrants using genetic data is not a trivial task and requires a thorough characterization of type I and II errors associated with it. Application of genome-wide approaches in this context would certainly be beneficial since increased marker density enables a more reliable identification of migrants as well as hybrid genotypes [14].

To conclude, we appreciate the incorporation of different types of evidence to advance science, and the work by Garcia *et al*. [1] represents an interesting attempt to address a long-standing conundrum in fish ecology [15]. However, a more careful evaluation of the empirical knowledge supporting human- and avian-mediated transportation of fish is needed, together with appreciation of the existing methodological limitations, to solve the mind-boggling question of how perch colonizes islands of water surrounded by dry land.

## Data Availability

Electronic supplementary material includes the results of genetic assignment simulations performed using ONCOR software [13]. The data are provided in the electronic supplementary material [16].
